# Association between metabolic dysfunction-associated steatotic liver disease and risk of colorectal cancer or colorectal adenoma: an updated meta-analysis of cohort studies

**DOI:** 10.3389/fonc.2024.1368965

**Published:** 2024-07-09

**Authors:** Jian-Feng Zhao, Ben-Gang Zhou, Yang Lv, Qiu-Ping Teng, Xi-Mei Wang, Xiao-Yi Li, Yi Ding

**Affiliations:** ^1^ Department of Gastrointestinal Surgery, Jingmen People’s Hospital, Jingchu University of Technology Affiliated Central Hospital, Jingmen, Hubei, China; ^2^ Dalian Medical University, Dalian, Liaoning, China; ^3^ Department of Nephrology, The Central Hospital of Jingmen, Jingmen, Hubei, China; ^4^ Imaging Diagnosis Center, Jingmen People’s Hospital, Jingchu University of Technology Affiliated Central Hospital, Jingmen, Hubei, China

**Keywords:** metabolic dysfunction-associated steatotic liver disease, non-alcoholic fatty liver disease, colorectal cancer, colorectal adenoma, meta-analysis, systematic review, cohort studies

## Abstract

**Background and aims:**

In recent years, the relationship between metabolic dysfunction-associated steatotic liver disease (MASLD) and colorectal cancer (CRC) or colorectal adenoma (CRA) has gained widespread attention. Previous meta-analyses on this subject either incorporated numerous cross-sectional studies, which were susceptible to bias, or concentrated solely on a restricted number of cohort studies. Moreover, with the release of a substantial number of high-quality cohort studies on this subject in the past two years, the findings continue to be debated and contradictory. Therefore, we conducted an updated systematic review and meta-analysis of cohort studies to quantitatively evaluate the magnitude of the association between them.

**Methods:**

Comprehensive searches of PubMed, Web of Science, and Embase were conducted without language restrictions from the time of their creation up to December, 2023. The pooled hazard ratios (HRs) with 95% confidence interval (CIs) were calculated by the generic inverse variance based on the random-effects model. Moreover, subgroup and sensitivity analyses were performed.

**Results:**

A total of 15 cohort studies were analyzed in this meta-analysis, which included 9,958,412 participants. The meta-analysis of 13 cohort studies showed that MASLD was linked to a higher risk of CRC (HR=1.25, 95% CI: 1.15-1.36, *P* < 0.00001). Additionally, further subgroup analysis indicated that the combined HR remained consistent regardless of the study location, nomenclature of fatty liver disease (FLD), confirmation methods for FLD, sample size, follow-up time, and study quality. Furthermore, the meta-analysis of four cohort studies demonstrated that MASLD was correlated with an increased risk of CRA (HR=1.38, 95% CI: 1.17-1.64, *P* = 0.0002). The sensitivity analysis results further validated the robustness of the aboved findings.

**Conclusion:**

The results of our meta-analysis indicated that MASLD was associated with an increased risk of incident CRC/CRA. In the future, it is necessary to conduct more prospective cohort studies to thoroughly assess potential confounding factors, particularly in individuals from Europe and North America. Furthermore, related mechanism studies should be conducted to enhance our understanding of the link between MASLD and CRC/CRA.

**Systematic review registration:**

Open Science Framework registries (https://osf.io/m3p9k).

## 1 Introduction

Metabolic dysfunction-associated steatotic liver disease (MASLD), previously referred to as non-alcoholic fatty liver disease (NAFLD) or metabolic dysfunction-associated fatty liver disease (MAFLD), is the most prevalent chronic liver disease globally, affecting up to 30% of adults and 69.99% of overweight and 75.27% of obese populations (1–3). The prevalence of MASLD is on the rise in all age groups worldwide (4). The disease spectrum of MASLD includes hepatic steatosis, characterized by excessive accumulation of liver cell lipids, as well as more severe forms such as metabolic dysfunction-related steatohepatitis (MASH), liver fibrosis, cirrhosis, and liver cancer (1, 5). The growing body of evidence suggests that MASLD is a multi-system disease, causing not only serious liver-related complications, but also potentially leading to a variety of extrahepatic diseases, such as cardiovascular disease, diabetes, and extrahepatic cancer (6–9).

Colorectal cancer (CRC) ranks as the third most prevalent malignant tumor globally and has the second highest mortality rate (10). With the anticipated increase in CRC cases in the coming decades, there is expected to be a greater burden on global public health (10, 11). CRC typically arises from colorectal adenoma (CRA) following a lengthy premalignant phase. Despite the availability of chemotherapy, radiotherapy, surgical excision, immune checkpoint inhibitors, and targeted therapy, these treatment options do not offer an optimal solution for CRC patients (12). Hence, the identification of new and potential modifiable risk factors and the prevention of CRC have significant public health implications.

In recent years, with the introduction of the concept of “liver-gut axis”, the relationship between MASLD and the risk of CRC/CRA has gained widespread attention. MASLD and CRC/CRA shared common risks such as insulin resistance and metabolic syndrome (13). The question of whether MASLD is linked to a higher risk of CRC/CRA remains unresolved. Previously, several meta-analyses (14–22) have explored the association between MASLD and risk of CRC/CRA. Nevertheless, previous meta-analyses (14–22) comprised a significant number of cross-sectional studies that were more vulnerable to bias, with only a limited number of cohort studies included. Cohort studies are the most important and optimal type of research for investigating the causal relationship of this non-interference association. Moreover, with the release of a substantial number of high-quality cohort studies on this subject in the past two years, the findings continue to be debated and contradictory (23–27). Hence, we carried out an updated systematic review and meta-analysis of cohort studies to thoroughly outline the association between MASLD and CRC/CRA risk, offering insights for the prevention and screening of CRC/CRA.

## 2 Materials and methods

### 2.1 Registration of protocol and reporting guidelines

We have already registered our study protocol on Open Science Framework (https://osf.io/m3p9k). This study adhered to the Meta-analysis Of Observational Studies in Epidemiology (MOOSE) proposal (28) and the Preferred Reporting Items for Systematic Reviews and Meta-Analyses (PRISMA) reporting guidelines (29).

### 2.2 Literature search strategy

Comprehensive searches of PubMed, Web of Science, and Embase were conducted without language restrictions from the time of their creation up to December, 2023. The main search formulas were as follows: (“metabolic dysfunction-associated fatty liver disease” OR “metabolic associated fatty liver disease” OR “metabolic dysfunction-associated steatotic liver disease” OR “non-alcoholic fatty liver disease” OR “non-alcoholic fatty liver” OR “non-alcoholic steatohepatitis”) AND (“colorectal neoplasms” OR “colonic neoplasms” OR “rectal neoplasms” OR “colorectal cancer*” OR “colon cancer*” OR “rectal cancer*” OR “rectum cancer*” OR “colorectal tumour*” OR “colorectal tumor*” OR “colorectal carcinoma*” OR “colonic carcinoma*” OR “colon carcinoma*” OR “rectal carcinoma*” OR “rectum carcinoma*” OR “colorectal adenoma*” OR “colonic adenoma*” OR “colon adenoma*” OR “rectal adenoma*” OR “rectum adenoma*”). Taking into account the features of different databases, we conducted an all-encompassing search, combining Medical Subject Heading terms and text word searching. We examined all relevant references from original research and review articles to ensure the thoroughness of our search.

### 2.3 Study selection

Eligibility criteria were defined based on the following criteria: (1) cohort studies assessing the relationship between MASLD and the risk of CRC or CRA; (2) the exposure factor was MASLD; (3) the outcome was the risk of developing CRC or CRA among patients with MASLD compared to those without MASLD; (4) studies provided hazard ratios (HRs), relative risks (RRs), odds ratios (ORs), or incidence rate ratios (IRRs) with corresponding 95% confidence intervals (CIs), or adequate data for their calculation; (5) If multiple studies come from the same cohort, we will only choose the study with the longest follow-up time and a larger sample size. Studies were excluded based on the following criteria: (1) duplicated articles, letters, editorials, comments, abstracts, meta-analyses and reviews; (2) cross-sectional or case-control studies; (3) Studies without control group; (4) studies that cannot obtain relevant data by various ways. All studies that fulfilled the inclusion and exclusion criteria were independently scrutinized by two researchers (Zhao JF and Zhou BG), and any disagreements were settled through mutual consent.

### 2.4 Data extraction

Two independent researchers (Zhao JF and Lv Y) conducted the data extraction. The researchers both gathered the same information, such as the surname of the first author, the publication year, the study design, the study location, source of study subjects, the sample size, the population characteristics, the diagnostic techniques for MASLD and CRC or CRA, the follow-up duration, the HRs/RRs/ORs/IRRs with their 95% CIs, and the adjusted confounding factors. If any discrepancies arose concerning the extracted data, a third investigator was consulted to resolve the conflict.

### 2.5 Methodological quality assessment

The Newcastle-Ottawa Scale (NOS) (30) was utilized to assess the methodological quality of the studies included. This scale assesses a study on three domains, providing up to four stars for participant selection, two stars for the comparability of study groups, and three stars for the determination of outcomes of interest; thus, a maximum of nine stars can be achieved. We deem studies with a score of 8 or 9 stars to be of high quality, those with a score of 6 or 7 stars to be of medium quality, and those with a score of 5 stars or less to be of low quality (31).

### 2.6 Statistical analysis

The pooled HRs with 95% CIs were calculated by the generic inverse variance based on the random-effects model, which takes into account any discrepancies among studies, even if there are no statistically significant heterogeneity (32). Due to the rarity of the outcome of interest, the risk estimations of ORs, HRs, IRRs, and RRs were equal (33). For studies with more than one adjustment model, we chose the models that accounted for the largest number of potential confounding factors. The Cochran’s Q-test and I² statistic were employed to evaluate the statistical heterogeneity, with a *P* ≤ 0.10. I^2^ values of 0-25% are indicative of insignificant heterogeneity, whereas values from 26-50% represent low heterogeneity, 51-75% show moderate heterogeneity, and 76-100% demonstrate high heterogeneity (34). Subgroup analyses were conducted taking into account factors such as study location, nomenclature of fatty liver disease (FLD), confirmation methods for FLD, sample size, follow-up time, and study quality, to evaluate the uniformity of the pooled results and recognize any potential sources of heterogeneity. To determine the robustness of the results, a leave-one-out method (removing each individual study at a time and then combining the rest of the studies) was utilized to perform sensitivity analyses (31). To evaluate the potential publication bias, funnel plots and Begg’s (35) and Egger’s tests (36) were employed. A *P*-value of less than 0.05 was taken as an indicator of statistical significance. The Cochrane Collaboration’s Review Manager software 5.3 (Copenhagen, Denmark) and STATA/SE software (Version 12.0, STATA Corporation, Texas, USA) were both employed to carry out all statistical analyses.

## 3 Results

### 3.1 Study selection process

**Figure 1:**
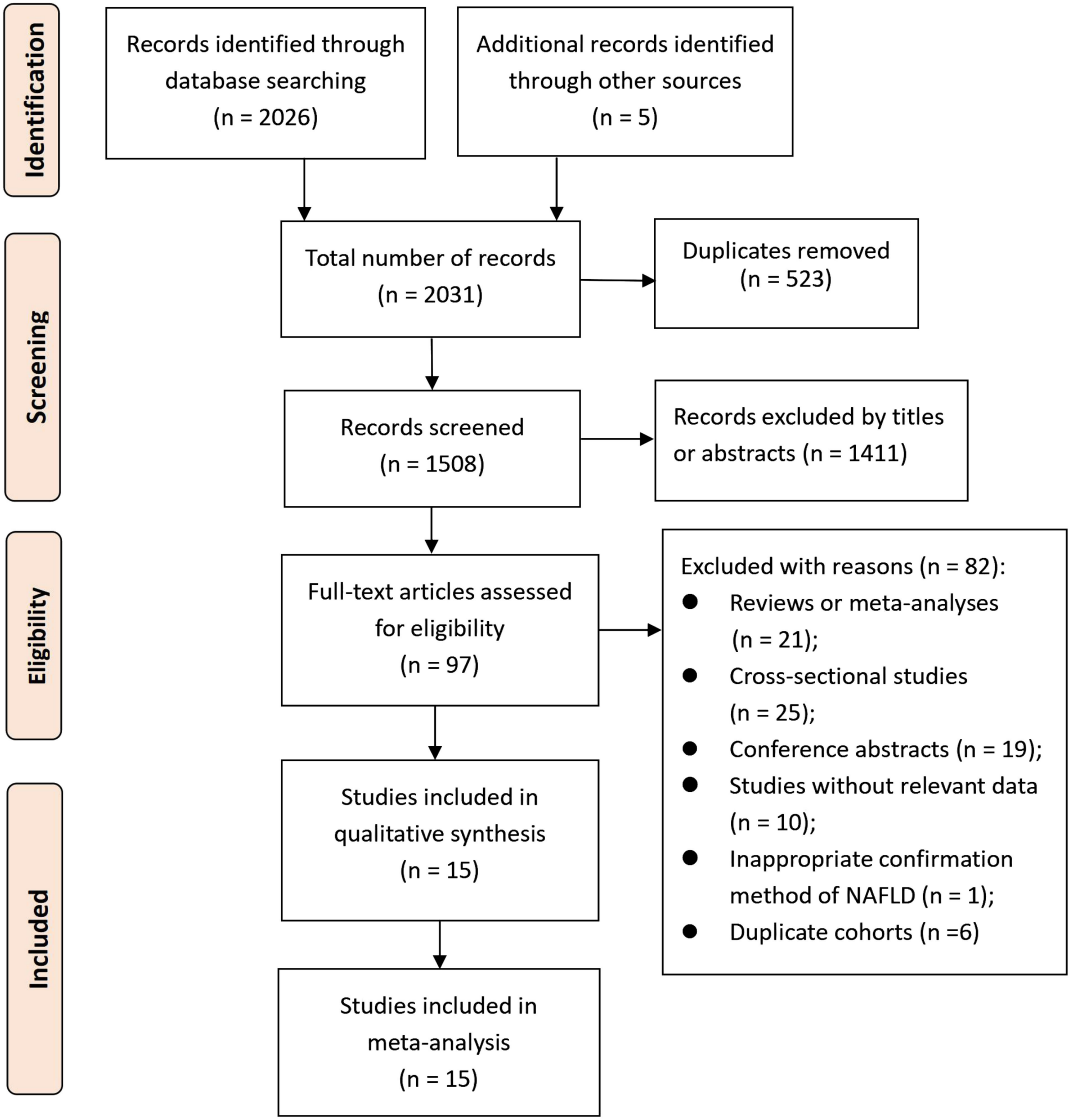
The PRISMA flowchart of study selection process.

A total of 2031 records were searched for screening, resulting in 1508 records after eliminating duplicates. Following the initial screening process, 1411 records were removed based on titles or abstracts. The 97 potential eligible records were then acquired and further evaluated, with 82 records being excluded for various reasons extensively described in **Supplementary Table S1**. As a result, 15 cohort studies (23–27, 37–46) met the criteria for inclusion in this meta-analysis. The process of selecting studies is depicted in **Figure 1**.

### 3.2 Study characteristics and quality assessment

A total of 9,958,412 participants were included in 15 cohort studies that were published between 2012 and 2023. The included studies were from various regions: eleven (24, 26, 27, 37–41, 43, 44, 46), from Asia (China, South Korea, Japan), three (23, 25, 45) from Europe (Sweden and the United Kingdom), and just one (42) from North America (United States). The sample sizes in the included studies varied from 1023 to 8,933,017 individuals. Among these 15 studies, 11 explored the relationship between MASLD and CRC (23–27, 39, 41–43, 45, 46), two (38, 40) explored the relationship between MASLD and CRA, and the remaining two (37, 44) explored the relationship between MASLD and both CRC and CRA simultaneously. Out of the 15 studies, three (24, 25, 27) utilized the terminology MAFLD, whereas the remaining seventeen studies employed the terminology NAFLD. The confirmation methods for FLD varied among the studies, with seven (27, 37, 38, 41, 43, 44, 46) utilizing ultrasonography (USG), two (24, 25) utilizing fatty liver index (FLI), four (23, 26, 39, 42) utilizing international classification of diseases (ICD) codes, one (40) utilizing USG or computer tomography (CT), and one (45) utilizing liver biopsy. With regard to the confirmation methods of CRC/CRA, five (38, 40, 41, 44, 45) utilized histopathology, one study (27) used medical records, and the remaining studies used ICD codes. The average follow-up time of included studies spanned from 2.6 to 13.8 years. In terms of methodological quality evaluation, seven studies were rated as high quality with a score of 8 or 9, eight studies were rated as medium quality with a score of 6 or 7, and none of the studies were rated as low quality. **Table 1** gives a summary of the basic characteristics of the studies that were included, while **Supplementary Table 2** delineates the adjusted confounding factors in those included studies.

**Table 1:** Basic characteristics of included studies.

Author, year	Country	Sample size	Mean age (years)	Male (%)	Confirmation of FLD	Confirmation of CRC/CRA	Follow-up time (mean years)	NOS score
Lee, 2012 (37)	South Korea	5,517	47	0	USG	ICD codes	4.5	7
Huang, 2013 (38)	China	1,522	54	60	USG	Histopathology	2.6	7
Sun, 2015 (39)	China	10,545	61	62	ICD codes	ICD codes	3.6	7
Yang, 2017 (40)	South Korea	1,023	55	51	USG or CT	Histopathology	5	6
Kim, 2018 (41)	South Korea	25,947	48	54	USG	Histopathology	7.5	8
Allen, 2019 (42)	USA	19,163	54	47	ICD codes	ICD codes	8	6
Hamaguchi, 2019 (43)	Japan	27,944	45	31	USG	ICD codes	6.5	7
Kim, 2021 (44)	South Korea	6,182	44	75	USG	Histopathology	3.4	7
Simon, 2021 (45)	Sweden	48,799	51	58	Liver biopsy	Histopathology	13.8	9
Yamamoto, 2021 (46)	Japan	30,172	44	32	USG	ICD codes	14	8
Björkström, 2022 (23)	Sweden	79,349	53	56	ICD codes	ICD codes	6	7
Lee, 2022 (24)	South Korea	8,933,017	50	49	FLI	ICD codes	10.1	9
Liu, 2022 (25)	UK	352,911	58	49	FLI	ICD codes	8.2	8
Wu, 2023 (26)	China	264,930	42	55	ICD codes	ICD codes	8.5	8
Yuan, 2023 (27)	China	151,391	50	81	USG	Medical records	12.6	8

FLD, fatty liver disease; CRC, colorectal cancer; CRA, colorectal adenoma; NOS, Newcastle-Ottawa Scale; ICD, international classification of diseases; USG, Ultrasonography; CT, Computer tomography; FLI, Fatty liver index; UK, United Kingdom.

### 3.3 Association between MASLD and risk of CRC

**Figure 2:**
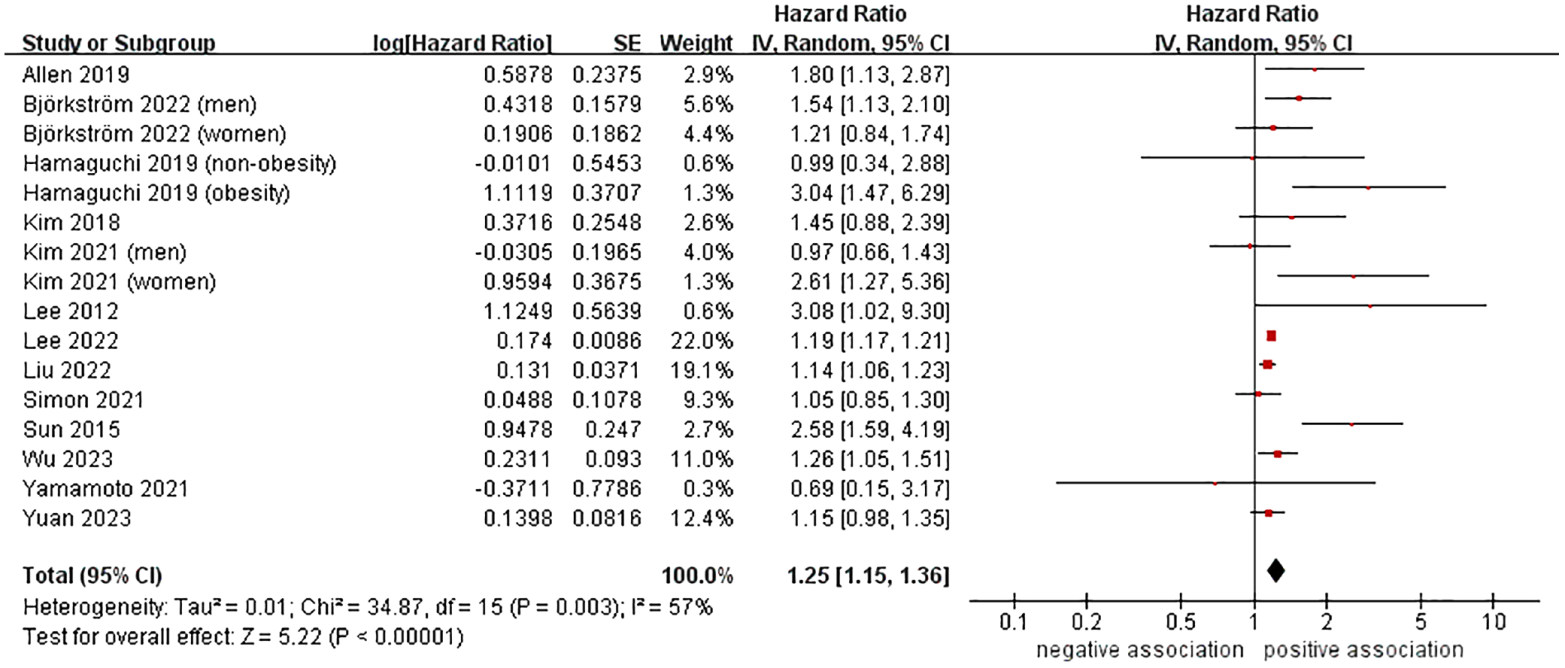
Forest plot of association between MASLD and the risk of incident CRC.

A total of 13 cohort studies (23–27, 37, 39, 41–46), involving 9,955,867 participants, investigated the association between MASLD and the likelihood of developing CRC. The meta-analysis revealed that MASLD was linked to a higher risk of incident CRC, with a pooled HR of 1.25 and a 95% CI of 1.15-1.36 (*P* < 0.00001). There was moderate heterogeneity (I^2 ^= 57%, *P* = 0.003) (**Figure 2**).

We conducted several subgroup analyses to investigate the potential sources of heterogeneity and factors that may impact the overall results. The results of subgroup analyses, which were based on study location, nomenclature of FLD, sample size, follow-up time, and study quality, were in line with the overall pooled results. In subgroup analysis stratified by confirmation of FLD, MASLD was found to be associated with an increased risk of incident CRC when USG (n = 6, HR = 1.45, 95% CI: 1.07-1.97, *P* = 0.02), FLI (n = 2, HR = 1.18, 95% CI: 1.15-1.22, *P* < 0.00001), and ICD codes (n = 4, HR = 1.52, 95% CI: 1.21-1.92, *P* = 0.0004) were used to confirm FLD. However, no significant association was observed between them when liver biopsy was used to confirm FLD (n = 1, HR = 1.05, 95% CI: 0.85-1.30, *P* = 0.65). The results of the subgroup analyses were shown in **Table 2**, as well as in **Supplementary Figures S1**-**S6**.

**Table 2:** Subgroup analyses on the association MAFLD and CRC.

Subgroups	No. of studies	HR (95%CI)	*P*_association_	*I^2^* (%)	*P*_heterogeneity_
Study location					
Asia	9	1.35 (1.16-1.58)	0.0001	62	0.003
Europe	3	1.17 (1.04-1.31)	0.009	30	0.23
North America	1	1.80 (1.13-2.87)	0.01	–	–
Nomenclature of FLD					
MAFLD	3	1.19 (1.17-1.21)	< 0.00001	0	0.49
NAFLD	10	1.47 (1.21-1.77)	< 0.0001	59	0.004
Confirmation of FLD					
USG	6	1.45 (1.07-1.97)	0.02	56	0.02
FLI	2	1.18 (1.15-1.22)	< 0.00001	22	0.26
ICD codes	4	1.52 (1.21-1.92)	0.0004	58	0.05
Liver biopsy	1	1.05 (0.85-1.30)	0.65	–	–
Sample size					
≥50,000	5	1.19 (1.17-1.21)	< 0.00001	0	0.47
<50,000	8	1.61 (1.18-2.19)	0.003	68	0.001
Follow-up time					
≥ 10 years	4	1.19 (1.17-1.21)	< 0.00001	0	0.57
< 10 years	9	1.47 (1.23-1.76)	< 0.0001	66	0.0006
Study quality					
Moderate-quality	6	1.69 (1.29-2.23)	0.0002	59	0.01
High-quality	7	1.19 (1.17-1.21)	< 0.00001	0	0.64
**Overall studies**	**13**	**1.25 (1.15-1.36)**	**< 0.00001**	**57**	**0.003**

FLD, fatty liver disease; MAFLD, Metabolic dysfunction-associated fatty liver disease; NAFLD, nonalcoholic fatty liver disease; CRC, colorectal cancer; HR, hazard ratio; CI, confidence interval; USG, Ultrasonography; FLI, Fatty liver index; ICD, International Classification of Diseases.Bold values indicates the results of overall meta-analysis.

Furthermore, we conducted a sensitivity analysis by removing each individual study and then combining the rest of the studies using the leave-one-out method. The findings showed that there were no significant changes in the results when any of the studies were excluded, suggesting that our results are robust (**Supplementary Table 3**).

With respect to publication bias assessment, the Begg’s funnel plot displayed a slightly asymmetric distribution (see **Supplementary Figures S7**). However, the Begg’s test and Egger’s test showed no evidence of significant publication bias (*P*_Begg_ = 0.115, *P*_Egger_ = 0.082).

### 3.4 Association between MASLD and risk of CRA

**Figure 3:**
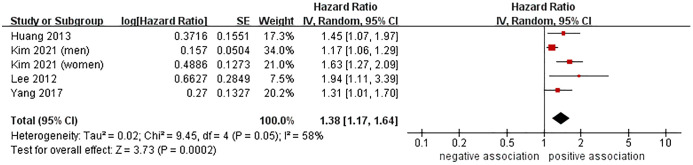
Forest plot of association between MASLD and the risk of incident CRA.

Data from 4 cohort studies (37, 38, 40, 44) involving 14,244 participants was analyzed to investigate the relationship between MASLD and the risk of CRA occurrence. The meta-analysis indicated a significant association between MASLD and the heightened risk of incident CRA (HR = 1.38, 95% CI: 1.17-1.64, *P* = 0.0002), accompanied by moderate heterogeneity (I^2^ = 58%, *P* = 0.05) (**Figure 3**). Moreover, the results of sensitivity analyses using the leave-one-out method had no impact on the overall findings, indicating the stability of the results (**Supplementary Table 4**). We chose not to conduct further publication bias testing due to the limited number of studies included.

## 4 Discussion

### 4.1 Principal findings

This meta-analysis gathered available evidence from 15 cohort studies, encompassing 9,958,412 participants across Asia, Europe, and North America, in order to investigate the association between MASLD and the likelihood of developing CRC/CRA. The meta-analysis of data from 13 cohort studies showed that MASLD was linked to a higher risk of incident CRC (HR=1.25, 95% CI: 1.15-1.36, *P* < 0.00001). Additionally, further subgroup analysis indicated that the combined HR remained consistent regardless of the study location, the terminology used for fatty liver disease (FLD), confirmation methods for FLD, sample size, follow-up time, and study quality. Furthermore, the meta-analysis of data from four cohort studies demonstrated that MASLD was correlated with an increased risk of incident CRA (HR=1.38, 95% CI: 1.17-1.64, *P* = 0.0002). The sensitivity analysis results further validated the robustness of the aboved findings.

### 4.2 Comparison with previous studies

To our knowledge, this meta-analysis is the most recent, largest, and most comprehensive research to date on the association between MASLD and the risk of incident CRC/CRA (focusing only on cohort studies). Previously, there were two meta-analyses (14, 15) were conducted that specifically examined the relationship between MASLD and CRA. In 2014, Shen et al. (14) performed a smaller meta-analysis of five observational studies (one cohort and four cross-sectional studies) and found that MASLD was significantly linked to CRA with a pooled OR of 1.74 and a 95% CI of 1.53-1.97. The association was more pronounced in the Asian population (n=3) with a pooled OR of 1.77 and a 95% CI of 1.52-2.05, compared to the European or North American population (n=2) with a pooled OR of 1.42 and a 95% CI of 0.75-2.67. Similar results were observed in another meta-analysis in 2015 (n = 5, OR = 1.56, 95% CI: 1.22-1.99, *P* = 0.0003). In 2018, Mantovani et al. (16) conducted an updated meta-analysis (including 11 observational studies), and this meta-analysis indicated that MASLD was linked to a higher risk of prevalent CRC/CRA (n= 8 cross-sectional studies, OR = 1.40, 95% CI: 1.24-1.57; I^2^ = 78.8%) and incident CRC/CRA (n= 3 cohort studies, HR = 1.47, 95% CI: 1.20-1.81; I^2^ = 11.5%). Subsequently, Chen et al. (17) performed a meta-analysis and showed that MASLD was linked to a higher risk of developing any incident CRA (n = 9, adjusted OR: 1.30, 95% CI:1.19-1.43) and advanced incident CRA/CRC (n = 8, adjusted OR: 1.57, 95% CI: 1.21-2.04). Furthermore, the meta-analysis also found that severe MASLD was associated with a greater risk of incident CRA/CRC compared to mild and/or moderate MASLD (n = 5, adjusted OR: 2.19, 95% CI: 1.33-3.60). In 2020, another larger meta-analysis (18) of 20 observational studies investigated the relationship MASLD and colorectal polyps (including unclassified colorectal polyps, hyperplastic polyps, adenomas, and cancers), and reached similar conclusions. In 2021 and 2022, Lin et al. (19) and Ye et al. (20) conducted two other meta-analyses, which included 11 studies (9 cross-sectional studies and 2 cohort studies) and 14 studies (7 cross-sectional studies and 7 cohort studies), respectively. Both of these meta-analyses demonstrated a connection between MASLD and a higher risk of CRC/CRA. Similar results were observed in another two meta-analyses (21, 22). Recently, Mantovani et al. (31) also conducted a larger meta-analysis of 10 cohort studies (published between 1996 and 2020) with 182,202 middle-aged individuals and indicated that MASLD was found to be linked with a nearly 60% increased risk of CRC (n = 8, HR = 1.64, 95% CI: 1.24-2.19, I^2^ = 58%) and a 40% higher risk of CRA (n = 4, HR = 1.40, 95% CI: 1.20-1.63, I^2^ = 30%).

In comparison to all previous meta-analyses, our most recent meta-analysis not only validates but also expands upon their findings. Firstly, our meta-analysis includes larger total sample size (n = 9,958,412) and provides high statistical power to quantitatively assess the relationship between MASLD and CRC/CRA. Secondly, our updated meta-analysis encompasses all cohort studies included in previous meta-analyses, while excluding a large number of cross-sectional studies that are more susceptible to bias. It also includes high-quality cohort research evidence published from 2022 to 2023, providing the latest, most comprehensive, and most reliable evidence for this topic. Thirdly, all prior meta-analyses solely incorporated studies on the relationship between the term NAFLD and CRC/CRA. In contrast, our meta-analysis encompassed four studies on the correlation between the term MAFLD (a new nomenclature) and CRC/CRA. Considering the slight differences in diagnostic criteria between NAFLD and MAFLD, our meta-analysis contributed to strengthen the existing research evidence on this subject.

### 4.3 Potential explanations and implications

The exact pathophysiological mechanism connecting NAFLD and CRC/CRA is not completely understood. It is uncertain whether the risk is solely due to shared metabolic risk factors, or if MASLD itself directly contributes to the development of CRC/CRA regardless of these shared factors. The close association between MASLD, abdominal obesity, diabetes, and insulin resistance (IR) makes it challenging to determine the exact causal relationship between MASLD and the increased risk of CRC/CRA. With the liver-gut axis concept gaining traction, it is becoming increasingly evident that the liver and intestines are linked by shared underlying pathophysiological pathways (13, 16). Several possible explanations have been proposed at present. Firstly, the role of IR may be crucial in the connection between two diseases. The occurrence and advancement of MASLD are significantly influenced by IR (47). Numerous studies have shown that IR and its related conditions, such as elevated insulin and insulin-like growth factor (IGF-1), can contribute to the onset and progression of CRC/CRA (48). Secondly, the inflammatory response could potentially have a significant impact on CRC/CRA, and MASLD has the potential to induce mild systemic inflammation, resulting in the production of pro-inflammatory cytokines like tumor necrosis factor alpha (TNF-α), interleukin (IL) -6, and IL-8. These pro-inflammatory factors are crucial in promoting cell proliferation, inhibiting cell apoptosis, and stimulating angiogenesis (13, 48–51). Thirdly, the carcinogenic process may involve a number of adipokines that are responsible for regulating metabolism, inflammation, and fiber formation. Adiponectin and leptin, which are secreted by adipocytes, play a crucial role in the connection between MASLD and CRC/CRA development. Patients with MASLD may experience alterations in adipocyte cytokine metabolism, leading to decreased levels of adiponectin and increased levels of leptin. This imbalance may disrupt the anticancer properties of leptin in the colon, while also potentially promoting carcinogenesis in the presence of low levels of adiponectin (13, 48, 52). Fourthly, the occurrence of MASLD and, CRC/CRA may be influenced by dysfunction of gut microbiota. MASLD is frequently linked to the disruption of the intestinal mucosal barrier, which can lead to dysbiosis of the gut microbiota. This can trigger a cascade of immune reactions in the intestinal mucosa, ultimately leading to intestinal inflammation and the promotion of CRC (53–55).

Our meta-analysis findings hold great clinical significance for healthcare professionals in managing patients with MASLD. It is important for doctors to be mindful of the potential cancer risks associated with MASLD and to closely monitor patients for early detection of CRC/CRA. Further large-scale prospective studies are needed to better understand the causal relationship between MASLD and CRC/CRA, taking into account factors such as race, diagnostic methods, MASLD severity, and potential confounding variables.

### 4.4 Strengths and limitations

The strengths of our study are demonstrated in several ways. Firstly, as previously stated, this meta-analysis is the most recent, comprehensive, and extensive research to date on the topic. The large sample size allows us to quantitatively and accurately examine the connection between MASLD and CRC/CRA. Secondly, through the implementation of multiple subgroup analyses and sensitivity analyses, we further established the credibility and stability of the research outcomes. Thirdly, all the cohort studies included are of medium to high quality, which ensures the reliability of the results.

Nevertheless, this study has certain limitations. First, while all the studies considered confounding factors, the specific adjustments made were inconsistent. Some studies did not fully address common risk factors for CRC/CRA, such as smoking, physical activity, body mass index, diabetes, and family history of CRC/CRA. Additionally, there may be residual and unmeasured confounding factors that could influence the accuracy of the results. Second, due to the lack of detailed information on the severity and treatment of MASLD in most studies, we are unable to further analyze the impact of these factors on the results, but we cannot completely rule them out. Third, our meta-analysis had a moderate to high degree of heterogeneity, which may compromise the reliability of our research findings. To mitigate this, we utilized a random effects model to account for the variations between studies. Despite conducting multiple subgroups and sensitivity analyses, the source of heterogeneity remains unidentified, suggesting that unknown or unmeasured confounding factors may be contributing to the heterogeneity. Fourth, most of the studies we included confirmed fatty liver using USG, FLI, and ICD codes, with only one study utilizing liver biopsy. Despite liver biopsy being widely accepted as the gold standard for diagnosing MASLD and evaluating liver fibrosis, it is generally not suitable for large-scale clinical studies. Fifth, although the NOS scores of the cohort studies we included are all of medium to high quality, it must be acknowledged that these ratings have a certain degree of subjectivity. Some studies still have certain methodological deficiencies such as population selection and comparability. In the future, further population-based, large-scale, high-quality cohort studies are needed to further verify the above findings. Finally, the majority of the cohort studies we included were from Asian countries, with only three studies from Europe and one study from the United States. Consequently, this conclusion mainly applies to the Asian population. Considering the differences in body fat distribution, genetic/cultural background, and lifestyle habits between Asian and non-Asian populations, which may have a significant impact on the development of CRC/CRA, it is essential to conduct larger scale, high-quality prospective cohort studies in European and American populations to further validate the above findings.

## 5 Conclusions

The results of our meta-analysis indicated that MASLD was associated with an increased risk of incident CRC/CRA. In the future, it is necessary to conduct more prospective cohort studies to thoroughly assess potential confounding factors, particularly in individuals from Europe and North America. Furthermore, related mechanism studies should be conducted to enhance our understanding of the link between MASLD and CRC/CRA.

## Data availability statement

The original contributions presented in the study are included in the article/**Supplementary Material**. Further inquiries can be directed to the corresponding author.

## Author contributions

JZ: Conceptualization, Data curation, Formal analysis, Investigation, Methodology, Project administration, Resources, Supervision, Validation, Visualization, Writing – original draft, Writing – review & editing. BZ: Conceptualization, Data curation, Formal analysis, Investigation, Methodology, Resources, Software, Supervision, Visualization, Writing – original draft, Writing – review & editing. YL: Conceptualization, Data curation, Formal analysis, Investigation, Writing – original draft. QT: Data curation, Supervision, Validation, Writing – original draft. XW: Data curation, Supervision, Validation, Writing – original draft. XL: Supervision, Validation, Writing – original draft. YD: Conceptualization, Investigation, Project administration, Resources, Supervision, Validation, Writing – original draft, Writing – review & editing.
