# Supplementary material for: Association between metabolic dysfunction-associated steatotic liver disease and risk of colorectal cancer or colorectal adenoma: an updated meta-analysis of cohort studies
Source: Front Oncol. 2024 Jul 9;14:1368965. doi: 10.3389/fonc.2024.1368965 (PMC11263091; doi:10.3389/fonc.2024.1368965)
Supplement: Supplementary file 1 [file DataSheet_1.pdf]

Supplementary online material

Association between metabolic dysfunction-associated steatotic liver disease and risk of colorectal cancer or colorectal adenoma: an updated meta-analysis of cohort studies

Table S1. Description of excluded studies

Table S2. The adjusted confounding factors of included studies

Table S3. Results of sensitivity analyses on the association between MASLD and CRC

Table S4. Results of sensitivity analyses on the association between MASLD and CRA

Figure S1. Forest plot of the association between MASLD and risk of CRC of subgroup analysis based on study location

Figure S2. Forest plot of the association between MASLD and risk of CRC of subgroup analysis based on nomenclature of FLD

Figure S3. Forest plot of the association between MASLD and risk of CRC of subgroup analysis based on confirmation of FLD

Figure S4. Forest plot of the association between MASLD and risk of CRC of subgroup analysis based on sample size

Figure S5. Forest plot of the association between MASLD and risk of CRC of subgroup analysis based on follow-up time

Figure S6. Forest plot of the association between MASLD and risk of CRC of subgroup analysis based on study quality

Figure S7. The Begg's funnel plot for the association between MASLD and risk of CRC

Table S1. Description of excluded studies

No.	First author	Publication year	Reason for exclusion
1.	Hwang	2010	Cross-sectional study
2.	Stadlmayr	2011	Cross-sectional study
3.	Touzin	2011	Cross-sectional study
4.	Wong	2011	Cross-sectional study
5.	Wong	2011	Conference Abstract
6.	Arase	2012	Without relevant data
7.	Shen	2013	Cross-sectional study (conference Abstract)
8.	Zheng	2013	Conference Abstract (case-control study)
9.	Kondo	2014	Without relevant data
10	Lin	2014	Cross-sectional study
11	Shen	2014	Systematic review and meta-analysis (conference Abstract)
12	Shen	2014	Cross-sectional study (conference Abstract)
13	Basyigit	2015	Conference Abstract
14	Basyigit	2015	Cross-sectional study
15	Bhatt	2015	Cross-sectional study
16	Ding	2015	Systematic review and meta-analysis
17	Siow	2015	Conference Abstract
18	Tao	2015	Cross-sectional study
19	You	2015	Without relevant data
20	Ahn	2016	Cross-sectional study (conference Abstract)
21	Wong	2016	Without relevant data
22	Ahn	2017	Cross-sectional study
23	Chen	2017	Cross-sectional study
24	Ehrmann	2017	Review
25	Gravito-Soares	2017	Conference Abstract
26	Kim	2017	Duplicate conference abstract

27	Mikolasevic	2017	Review
28	Pan	2017	Cross-sectional study
29	Chen	2018	Without relevant data
30	Hicks	2018	Conference Abstract
31	Kim	2018	Conference Abstract
32	Mantovani	2018	Systematic review and meta-analysis
33	Verheyen	2018	Conference Abstract
34	Yu	2018	Cross-sectional study
35	Ahmed	2019	Review
36	Albhaisi	2019	Conference Abstract
37	Amalou	2019	Conference Abstract
38	Chen	2019	Systematic review and meta-analysis
39	Cho	2019	Cross-sectional study
40	Kim	2019	Conference Abstract
41	Kim	2019	Cross-sectional study
42	Li	2019	Cross-sectional study
43	Parizadeh	2019	Review
44	Blackett	2020	Cross-sectional study
45	Cai	2020	Conference Abstract
46	Chen	2020	Systematic review and meta-analysis
47	Lesmana	2020	Cross-sectional study
48	Liu	2020	Systematic review and meta-analysis
49	Patel	2020	Conference Abstract
50	Reja	2020	Conference Abstract
51	Yang	2020	Cross-sectional study
52	Huang	2021	Review
53	Lin	2021	Review
54	Lin	2021	Systematic review and meta-analysis

55	Seo	2021	Cross-sectional study
56	Veracruz	2021	Systematic review and meta-analysis
57	Yassine	2021	Conference Abstract
58	Zhang	2021	Meta-analysis
59	Zhang	2021	Without relevant data
60	Albhaisi	2022	Without relevant data
61	Fujii	2022	Conference Abstract
62	Gumussoy	2022	Without relevant data
63	Hagström	2022	Review
64	Mantovani	2022	Systematic review and meta-analysis
65	McHenry	2022	Inappropriate confirmation method of NAFLD
66	Qiu	2022	Without relevant data
67	Thomas	2022	Systematic review and meta-analysis
68	Ye	2022	Review
69	Zeng	2022	Systematic review and meta-analysis
70	Abu-Freha	2023	Conference Abstract
71	Afzaal	2023	Conference Abstract
72	Chang	2023	Cross-sectional study
73	Gong	2023	Cross-sectional study
74	Yang	2023	Cross-sectional study
75	Souza	2024	Systematic review and meta-analysis
76	Wei	2023	Without relevant data
77	Choi	2020	Duplicate cohort (from Korea National Health Insurance Service)
78	Lee	2020	Duplicate cohort (from Korea National Health Insurance Service)
79	Chuang	2023	Duplicate cohort (from Korea National Health Insurance Service)
80	Park	2023	Duplicate cohort (from Korea National Health Insurance Service)
81	Wang	2021	Duplicate cohort (from China Kailuan cohort)
82	Lan	2022	Duplicate cohort (from China Kailuan cohort)

References for the table

1. Hwang ST, Cho YK, Park JH, et al. Relationship of non-alcoholic fatty liver disease to colorectal adenomatous polyps. Journal of Gastroenterology and Hepatology. 2010;25(3):562-567.

2. Stadlmayr A, Aigner E, Steger B, et al. Nonalcoholic fatty liver disease: an independent risk factor for colorectal neoplasia. Journal of Internal Medicine. 2011;270(1):41-49.

3. Touzin NT, Bush KNV, Williams CD, Harrison SA. Prevalence of colonic adenomas in patients with nonalcoholic fatty liver disease. Therapeutic Advances in Gastroenterology. 2011;4(3):169-176.

4. Wong VW, Wong GL, Tsang SW, et al. High prevalence of colorectal neoplasm in patients with non-alcoholic steatohepatitis. Gut. 2011;60(6):829-836.

5. Wong VWS, Wong GLH, Tsang SWC, Fan T, Sung JJY, Chan HLY. High prevalence of advanced colorectal neoplasms in patients with nonalcoholic steatohepatitis: A prospective study. Hepatology International. 2011;5(1):188.

6. Arase Y, Kobayashi M, Suzuki F, et al. Difference in malignancies of chronic liver disease due to non-alcoholic fatty liver disease or hepatitis C in Japanese elderly patients. Hepatology Research. 2012;42(3):264-272.

7. Shen H, Lipka S, Zheng X, Rizvon K, Mustacchia P. The relationship between colorectal adenomas and fatty liver disease detected on imaging. American Journal of Gastroenterology. 2013;108:S186.

8. Zheng MH, Lin XF, Shi KQ, et al. Increased risk of colorectal malignant neoplasm in patients with nonalcoholic fatty liver disease: A large case-control study. Journal of Hepatology. 2013;58:S555.

9. Kondo T, Okabayashi K, Hasegawa H, et al. The impact of hepatic steatosis on the incidence of liver metastasis from colorectal cancer. Diseases of the Colon and Rectum. 2014;57(5):e293.

10. Lin XF, Shi KQ, You J, et al. Increased risk of colorectal malignant neoplasm in patients with nonalcoholic fatty liver disease: a large study. Mol Biol Rep. 2014;41(5):2989-2997.

11. Shen H, Lipka S, Kumar A, Mustacchia P. Association between nonalcoholic fatty liver disease and colorectal adenoma: A systemic review and meta-analysis. American Journal of Gastroenterology. 2014;109:S158-S159.

12. Shen H, Lipka S, Mustacchia P. The relationship between nonalcoholic fatty liver disease and colorectal adenomas. Gastroenterology. 2014;146(5):S-709.

13. Basyigit S, Kefeli A, Aktas B, et al. Relationship between insulin resistance non alcoholic fatty liver disease and colorectal carcinoma. Hepatology International. 2015;9(1):S371.

14. Basyigit S, Uzman M, Kefeli A, et al. Absence of non-alcoholic fatty liver disease in the presence of insulin resistance is a strong predictor for colorectal carcinoma. International Journal of Clinical and Experimental Medicine. 2015;8(10):18601-18610.

15. Bhatt BD, Lukose T, Siegel AB, Brown RS, Verna EC. Increased risk of colorectal polyps in patients with non-alcoholic fatty liver disease undergoing liver transplant evaluation. Journal of Gastrointestinal Oncology. 2015;6(5):459-468.

16. Ding W, Fan J, Qin J. Association between nonalcoholic fatty liver disease and colorectal adenoma: A systematic review and meta-analysis. International Journal of Clinical and Experimental Medicine. 2015;8(1):322-333.

17. Siow W, Niblett S, King K, Yates Z, Lucock M, Veysey M. NAFLD fibrosis score predicts an increased risk of colorectal polyps. Journal of Gastroenterology and Hepatology (Australia). 2015;30:112-113.

18. Tao W, Li H, Gao F. Correlations between adiponectin, non-alcoholic fatty liver disease and colorectal adenoma. Chinese Journal of Gastroenterology. 2015;20(1):38-41.

19. You J, Huang S, Huang GQ, et al. Nonalcoholic fatty liver disease: A negative risk factor for colorectal cancer prognosis. Medicine (United States). 2015;94(5):e479.

20. Ahn JS, Sinn DH, Min YW, et al. The risk of colorectal neoplasia according to the presence and severity of non-alcoholic fatty liver disease. Hepatology. 2016;64(1):543A-544A.

21. Wong MC, Ching JY, Chan VC, et al. Screening strategies for colorectal cancer among patients with nonalcoholic fatty liver disease and family history. Int J Cancer. 2016;138(3):576-583.

22. Ahn JS, Sinn DH, Min YW, et al. Non-alcoholic fatty liver diseases and risk of colorectal neoplasia. Alimentary Pharmacology & Therapeutics. 2017;45(2):345-353.

23. Chen QF, Zhou XD, Sun YJ, et al. Sex-influenced association of non-alcoholic fatty liver disease with colorectal adenomatous and hyperplastic polyps. World Journal of Gastroenterology. 2017;23(28):5206-5215.

24. Ehrmann J, Aiglová K, Vrzalová D, Aiglová R, Konečný M, Procházka V. Extrahepatic complication of nonalcoholic fatty liver disease. Is it a causal association? Interni Medicina pro Praxi. 2017;19(2):72-77.

25. Gravito-Soares M, Gravito-Soares E, Gomes D, Simão A, Tomé L. Liver transient elastography in non-alcoholic fatty liver disease: Is there any predictive role in the development of colorectal polyps? United European Gastroenterology Journal. 2017;5(5):A623.

26. Kim GA, Lee HC, An J, Shim JH, Kim HK, Choe J. Risk of cancer in patients with nonalcoholic fatty liver disease: A hospital-based cohort study. Hepatology International. 2017;11(1):S62.

27. Mikolasevic I, Orlic L, Stimac D, Hrstic I, Jakopcic I, Milic S. Non-alcoholic fatty liver disease and colorectal cancer. Postgraduate Medical Journal. 2017;93(1097):153-158.

28. Pan S, Hong W, Wu W, et al. The relationship of nonalcoholic fatty liver disease and metabolic syndrome for colonoscopy colorectal neoplasm. Medicine (Baltimore) .2017;96(2):e5809.

29. Chen ZF, Dong XL, Huang QK, et al. The combined effect of non-alcoholic fatty liver disease and metabolic syndrome on colorectal carcinoma mortality: a retrospective in Chinese females. World Journal of Surgical Oncology. 2018;16(1):163.

30. Hicks SB, Mara K, Larson JJ, Therneau TM, Allen AM. The incidence of extrahepatic malignancies in nonalcoholic fatty liver disease (NAFLD). Hepatology. 2018;68:20A.

31. Kim MC, Park JG, Jang BI, et al. Liver fibrosis is associated with risk for colorectal adenoma in patients with non-alcoholic fatty liver disease. Hepatology International. 2018;12(2):S443.

32. Mantovani A, Dauriz M, Byrne CD, et al. Association between nonalcoholic fatty liver disease and

colorectal tumours in asymptomatic adults undergoing screening colonoscopy: a systematic review and meta-analysis. Metabolism-Clinical and Experimental. 2018;87:1-12.

33. Verheyen E, Castaneda D, Liu Y, Weisberg I. OUTCOMES IN MALIGNANCY AND NON-ALCOHOLIC FATTY LIVER DISEASE (NAFLD); A NATIONWIDE ANALYSIS. Gastroenterology. 2018;154(6):S-1171.

34. Yu X, Xie L, Zhou Y, et al. Analysis of Biological Characteristics of Colorectal Polyps in Patients With Non-alcoholic Fatty Liver Disease. Chinese Journal of Gastroenterology. 2018;23(7):410-415.

35. Ahmed OT, Allen AM. Extrahepatic Malignancies in Nonalcoholic Fatty Liver Disease. Current hepatology reports. 2019;18(4):455-472.

36. Albhaisi S, Sanyal AJ. EXTRAHEPATIC NEOPLASMS IN PATIENTS WITH NONALCOHOLIC FATTY LIVER DISEASE. Gastroenterology. 2019;156(6):S-1262.

37. Amalou K, Belghanem F, Djamel K, et al. High prevalence of colonic adenom as in patients with nonalcoholic fatty liver disease: Algerian prospective study. United European Gastroenterology Journal. 2019;7(8):468-469.

38. Chen J, Bian D, Zang S, et al. The association between nonalcoholic fatty liver disease and risk of colorectal adenoma and cancer incident and recurrence: a meta-analysis of observational studies. Expert Review of Gastroenterology and Hepatology. 2019;13(4):385-395.

39. Cho Y, Lim SK, Joo SK, et al. Nonalcoholic steatohepatitis is associated with a higher risk of advanced colorectal neoplasm. Liver International. 2019;39(9):1722-1731.

40. Kim MC, Jang BI, Kang MK, et al. The risk for colorectal adenoma is associated with liver fibrosis in patients with non-alcoholic fatty liver disease. Endoscopy. 2019;51(4):S210-S211.

41. Kim MC, Park JG, Jang BI, Lee HJ, Lee WK. Liver fibrosis is associated with risk for colorectal adenoma in patients with nonalcoholic fatty liver disease. Medicine. 2019;98(6):e14139.

42. Li Y, Liu S, Gao Y, et al. Association between NAFLD and Risk of Colorectal Adenoma in Chinese Han Population. J Clin Transl Hepatol. 2019;7(2):99-105.

43. Parizadeh SM, Parizadeh SA, Alizade-Noghani M, et al. Association between non-alcoholic fatty liver disease and colorectal cancer. Expert Review of Gastroenterology & Hepatology. 2019;13(7):633-641.

44. Blackett JW, Verna EC, Lebwohl B. Increased Prevalence of Colorectal Adenomas in Patients with Nonalcoholic Fatty Liver Disease: A Cross-Sectional Study. Digestive Diseases. 2020;38(3):222-230.

45. Cai X, Chen W, Jing X. High risk of colorectal polyps in male patients of nonalcoholic fatty liver disease: A meta-analysis of observational studies. Journal of Digestive Diseases. 2020;21(SUPPL 1):18.

46. Chen W, Wang M, Jing X, et al. High risk of colorectal polyps in men with non-alcoholic fatty liver disease: A systematic review and meta-analysis. Journal of Gastroenterology and Hepatology (Australia). 2020;35(12):2051-2065.

47. Lesmana CRA, Pakasi LS, Sudoyo AW, Krisnuhoni E, Lesmana LA. The Clinical Significance of Colon Polyp Pathology in Nonalcoholic Fatty Liver Disease (NAFLD) and Its Impact on Screening Colonoscopy in

Daily Practice. Can J Gastroenterol Hepatol. 2020;2020:6676294.

48. Liu SS, Ma XF, Zhao J, et al. Association between nonalcoholic fatty liver disease and extrahepatic cancers: a systematic review and meta-analysis. Lipids in Health and Disease. 2020;19(1):118.

49. Patel YA, Lee TH, Niedzwiecki D, Parish A, Muir AJ, Fisher DA. NAFLD WITH FIBROSIS IS ASSOCIATED WITH AN INCREASED RISK OF NON-HCC CANCER BURDEN: AN ANALYSIS OF PCORNET DATA. Gastroenterology. 2020;158(6):S-1421-S-1422.

50. Reja D, Kabaria S, Tawadros A, Pioppo L, Makar M, Rustgi V. Extra-hepatic gastrointestinal malignancies in NAFLD: nationwide inpatient sample 2016. Journal of Hepatology. 2020;73:S148.

51. Yang R, Chen Y, Chen X. Value of routine test for identifying colorectal cancer from patients with nonalcoholic fatty liver disease. BMC Gastroenterol. 2020 Jun 9;20(1):180.

52. Huang X, Yan X, Zhang M, Wu J, Chen J. Association between nonalcoholic fatty liver disease and colorectal neoplasms. Journal of Clinical Hepatology. 2021;37(12):2947-2950.

53. Lin HP, Zhang XR, Li GL, Wong GLH, Wong VWS. Epidemiology and Clinical Outcomes of Metabolic (Dysfunction)-associated Fatty Liver Disease. Journal of Clinical and Translational Hepatology. 2021;9(6):972-982.

54. Lin X, You F, Liu H, Fang Y, Jin S, Wang Q. Site-specific risk of colorectal neoplasms in patients with non-alcoholic fatty liver disease: A systematic review and meta-analysis. PLoS One. 2021;16(1):e0245921.

55. Seo JY, Bae JH, Kwak MS, et al. The Risk of Colorectal Adenoma in Nonalcoholic or Metabolic-Associated Fatty Liver Disease. Biomedicines. 2021;9(10):1401.

56. Veracruz N, Hameed B, Saab S, Wong RJ. The Association Between Nonalcoholic Fatty Liver Disease and Risk of Cardiovascular Disease, Stroke, and Extrahepatic Cancers. Journal of Clinical and Experimental Hepatology. 2021;11(1):45-81.

57. Yassine AA, Dahabra L, Abureesh M, Alshami M, Sasso R, Deeb L. Could non-alcoholic fatty liver disease be an additional predisposing factor for colorectal cancer? A large population-based study in the US. American Journal of Gastroenterology. 2021;116(SUPPL):S518-S519.

58. Zhang H, Liu L, Guo X. Association of nonalcoholic fatty liver disease with the risk of colorectal adenoma and colorectal cancer: A Meta - analysis. Journal of Clinical Hepatology. 2021;37(7):1619-1625.

59. Zhang X, Wong VW, Yip TC, et al. Colonoscopy and Risk of Colorectal Cancer in Patients With Nonalcoholic Fatty Liver Disease: A Retrospective Territory-Wide Cohort Study. Hepatol Commun. 2021;5(7):1212-1223.

60. Albhaisi S, McClish D, Kang L, Gal T, Sanyal AJ. Nonalcoholic fatty liver disease is specifically related to the risk of hepatocellular cancer but not extrahepatic malignancies. Front Endocrinol (Lausanne). 2022;13:1037211.

61. Fujii H, Takahashi H, Sumida Y. Hepatic and extrahepatic cancers in patients with non-alcoholic fatty liver disease: A multicenter registry-based cohort study. Hepatology International. 2022;16:S270.

62. Gumussoy M, Koc O, Karatas G, et al. Factors associated with the development of extrahepatic malignancy

in patients with nonalcoholic fatty liver disease: A single-center longitudinal study. European Journal of Gastroenterology and Hepatology. 2022;34(11):1172-1177.

63. Hagström H, Kechagias S, Ekstedt M. Risk for hepatic and extra-hepatic outcomes in nonalcoholic fatty liver disease. J Intern Med. 2022 Aug;292(2):177-189.

64. Mantovani A, Petracca G, Beatrice G, et al. Non-alcoholic fatty liver disease and increased risk of incident extrahepatic cancers: a meta-analysis of observational cohort studies. Gut. 2022;71(4):778-788.

65. McHenry S, Zong XY, Shi MY, et al. Risk of nonalcoholic fatty liver disease and associations with gastrointestinal cancers. Hepatology Communications. 2022;6(12):3299-3310.

66. Qiu T, Hu W, Rao Z, Fang T. The molecular basis of the associations between non-alcoholic fatty liver disease and colorectal cancer. Front Genet. 2022;13:1007337.

67. Thomas JA, Kendall BJ, Dalais C, Macdonald GA, Thrift AP. Hepatocellular and extrahepatic cancers in non-alcoholic fatty liver disease: A systematic review and meta-analysis. European Journal of Cancer. 2022;173:250-262.

68. Ye S, Liu Y, Zhang T, et al. Analysis of the correlation between non-alcoholic fatty liver disease and the risk of colorectal neoplasms. Front Pharmacol. 2022;13:1068432.

69. Zeng Y, Cao R, Tao Z, Gao Y. Association between the severity of metabolic dysfunction-associated fatty liver disease and the risk of colorectal neoplasm: a systematic review and meta-analysis. Lipids Health Dis. 2022;21(1):52.

70. Abu-Freha N, Cohen B, Gordon M, et al. Comorbidities and Malignancy among NAFLD Patients Compared to the General Population, A Nation-Based Study. Biomedicines. 2023;11(4):1110.

71. Afzaal T, Hudson D, Vaan B, Khan MQ, Qumosani K, Teriaky A. Gastrointestinal malignancies in hospitalized patients with non-alcoholic fatty liver disease (NAFLD): Analysis of the national inpatient sample (NIS). Canadian Liver Journal. 2023;6(1):179-180.

72. Chang J, Chang Y, Cho Y, Jung HS, Park DI, Park SK, et al. Metabolic-associated fatty liver disease is associated with colorectal adenomas in young and older Korean adults. Liver Int. 2023 Nov;43(11):2548-2559.

73. Gong Y, Kang J, Wang X, Zheng Y, Sui Y, Lu W. Increased detection rates of advanced colorectal adenoma in women with metabolic dysfunction-associated fatty liver disease. Heliyon. 2023;9(11):e22391.

74. Yang Y, Teng Y, Shi J, et al. Association of nonalcoholic fatty liver disease with colorectal adenomatous polyps and non-adenomatous polyps: A cross-sectional study. European Journal of Gastroenterology and Hepatology. 2023;35(12):1389-1393.

75. Souza M, Diaz I, Barchetta I, Mantovani A. Gastrointestinal cancers in lean individuals with non-alcoholic fatty liver disease: A systematic review and meta-analysis. Liver International. 2024;44(1):6-14.

76. Wei S, Hao Y, Dong X, Huang J, Huang K, Xie Y, Liu H, Wei C, Xu J, Huang W, Dong L, Yang J. The relationship between metabolic dysfunction-associated fatty liver disease and the incidence rate of extrahepatic cancer. Front Endocrinol (Lausanne). 2023;14:985858.

77. Choi YJ, Lee DH, Han KD. Association between high fatty liver index and development of colorectal

cancer: a nationwide cohort study with 21,592,374 Korean. Korean J Intern Med. 2020;35(6):1354-1363. 39.

78. Lee JM, Han K, Ko SH. The association between nonalcoholic fatty liver disease and esophageal, stomach, or colorectal cancer: National population-based cohort study. PLoS One. 2020;15(1):e0226351.

79. Chung GE, Yu SJ, Yoo JJ, Cho Y, Lee KN, Shin DW, et al. Differential risk of 23 site-specific incident cancers and cancer-related mortality among patients with metabolic dysfunction-associated fatty liver disease: a population-based cohort study with 9.7 million Korean subjects. Cancer Commun (Lond). 2023;43(8):863-876.

80. Park JH, Hong JY, Shen JJ, Han K, Park JO, Park YS, et al. Increased Risk of Young-Onset Digestive Tract Cancers Among Young Adults Age 20-39 Years With Nonalcoholic Fatty Liver Disease: A Nationwide Cohort Study. J Clin Oncol. 2023 Jun 20;41(18):3363-3373.

81. Wang Z, Zhao X, Chen S, Wang Y, Cao L, Liao W, et al. Associations Between Nonalcoholic Fatty Liver Disease and Cancers in a Large Cohort in China. Clin Gastroenterol Hepatol. 2021;19(4):788-796.e4.

82. Lan Y, Lu Y, Li J, Hu S, Chen S, Wang Y, et al. Outcomes of subjects who are lean, overweight or obese with nonalcoholic fatty liver disease: A cohort study in China. Hepatol Commun. 2022;6(12):3393-3405.

Table S2. The adjusted confounding factors of included studies		
**First author (year)**	**Study location**	**Adjusted confounding factors**
Lee (2012)	Asia	Age, body mass index, smoking, hypertension, dyslipidemia, and impaired fasting glucose/diabetes
Huang (2013)	Asia	Age, sex, body mass index, smoking, hypertension, diabetes, and metabolic syndrome
Sun (2015)	Asia	Age, sex, obesity, hypertension, diabetes, dyslipidemia, viral hepatitis, colon polyps, diagnostic procedures of esophagogastroduodenoscopy, colonoscopy, abdomen computed tomography, or ultrasonography
Yang (2017)	Asia	Age, sex, body mass index,smoking, hypertension, diabetes, use of aspirin, lipid-lowering agents and imaging for NAFLD diagnosis
Kim (2018)	Asia	Age, sex, smoking, hypertension, diabetes, lipids, and gamma-glutamyltransferase levels
Allen (2019)	North America	Age, sex, and obesity
Hamaguchi (2019)	Asia	Age, sex, obesity, smoking, diabetes, physical activity, and alcohol consumption
Kim (2021)	Asia	Age, sex, smoking, family history of CRC, use of NSAIDs, regular exercise, and baseline adenoma characteristics.
Simon (2021)	Europe	Age, sex, calendar year, county of residence, cardiovascular disease, diabetes, hypertension, dyslipidemia, obesity, endstage renal disease, family history of cancer at age <50 years, education (three groups plus missing category), the number of recorded hospital encounters in the year preceding the index biopsy date (or corresponding matching date), and alcohol abuse/misuse
Yamamoto (2021)	Asia	Age, sex, body mass index, smoking, dyslipidemia, diabetes, thyroid dysfunction, hypertension, sleep apnea, chronic kidney disease, cardiovascular disease

Björkström (2022)	Europe	Age, sex, diabetes, hypertension, hyperlipidaemia and chronic obstructive pulmonary disease
Lee (2022)	Asia	Age, sex, household income quartile, residential area Charlson Comorbidity Index, aspirin use, NSAID use, and tobacco
Liu (2022)	Europe	Age, sex, education, assessment center, household income, smoking status, pure alcohol intake, and physical activity
Wu (2023)	Asia	Age, sex, congestive heart failure, chronic kidney disease, chronic pulmonary disease, cerebrovascular disease, diabetes mellitus, human immunodeficiency virus, inflammatory bowel disease, myocardial infarction, and peripheral vascular disease
Yuan (2023)	Asia	Age, sex, education level, smoking status, alcohol consumption, physical activity, and family history of cancers
NAFLD, non-alcoholic fatty liver disease; CRC, colorectal cancer; NSAIDs, nonsteroidal anti-inflammatory drugs.		

Table S3. Results of sensitivity analyses on the association between MAFLD and CRC			
**Studies omitted**	**HR (95% CI)**	$P_{association}$	**Heterogeneity**
Lee (2012)	1.24 (1.14-1.35)	＜ 0.00001	I^2 = 56%, P = 0.004
Sun (2015)	1.21 (1.13-1.31)	＜ 0.00001	I^2 = 44%, P = 0.003
Kim (2018)	1.25 (1.15-1.36)	＜ 0.00001	I^2 = 59%, P = 0.002
Allen (2019)	1.24 (1.14-1.35)	＜ 0.00001	I^2 = 56%, P = 0.004
Hamaguchi (2019)	1.23 (1.14-1.33)	＜ 0.00001	I^2 = 54%, P = 0.008
Kim (2021)	1.25 (1.15-1.35)	＜ 0.00001	I^2 = 56%, P = 0.006
Simon (2021)	1.28 (1.17-1.40)	＜ 0.00001	I^2 = 58%, P = 0.002
Yamamoto (2021)	1.26 (1.15-1.37)	＜ 0.00001	I^2 = 59%, P = 0.002
Björkström (2022)	1.24 (1.13-1.36)	＜ 0.00001	I^2 = 60%, P = 0.002
Lee (2022)	1.33 (1.17-1.52)	＜ 0.0001	I^2 = 60%, P = 0.002
Liu (2022)	1.32 (1.18-1.49)	＜ 0.00001	I^2 = 58%, P = 0.002
Wu (2023)	1.26 (1.15-1.38)	＜ 0.00001	I^2 = 59%, P = 0.002
Yuan (2023)	1.28 (1.16-1.41)	＜ 0.00001	I^2 = 60%, P = 0.002
MAFLD, Metabolic dysfunction-associated fatty liver disease; CRC, colorectal cancer.			

Table S4. Results of sensitivity analyses on the association between MAFLD and CRA			
**Studies omitted**	**HR (95% CI)**	$P_{association}$	**Heterogeneity**
Lee (2012)	1.34 (1.14-1.58)	0.0005	I^2 = 58%, P = 0.07
Huang (2013)	1.38 (1.13-1.70)	0.002	I^2 = 65%, P = 0.04
Yang (2017)	1.43 (1.14-1.79)	0.002	I^2 = 68%, P = 0.02
Kim (2021)	1.42 (1.18-1.71)	0.0002	I^2 = 0%, P = 0.45
MAFLD, Metabolic dysfunction-associated fatty liver disease; CRA, colorectal adenoma.			

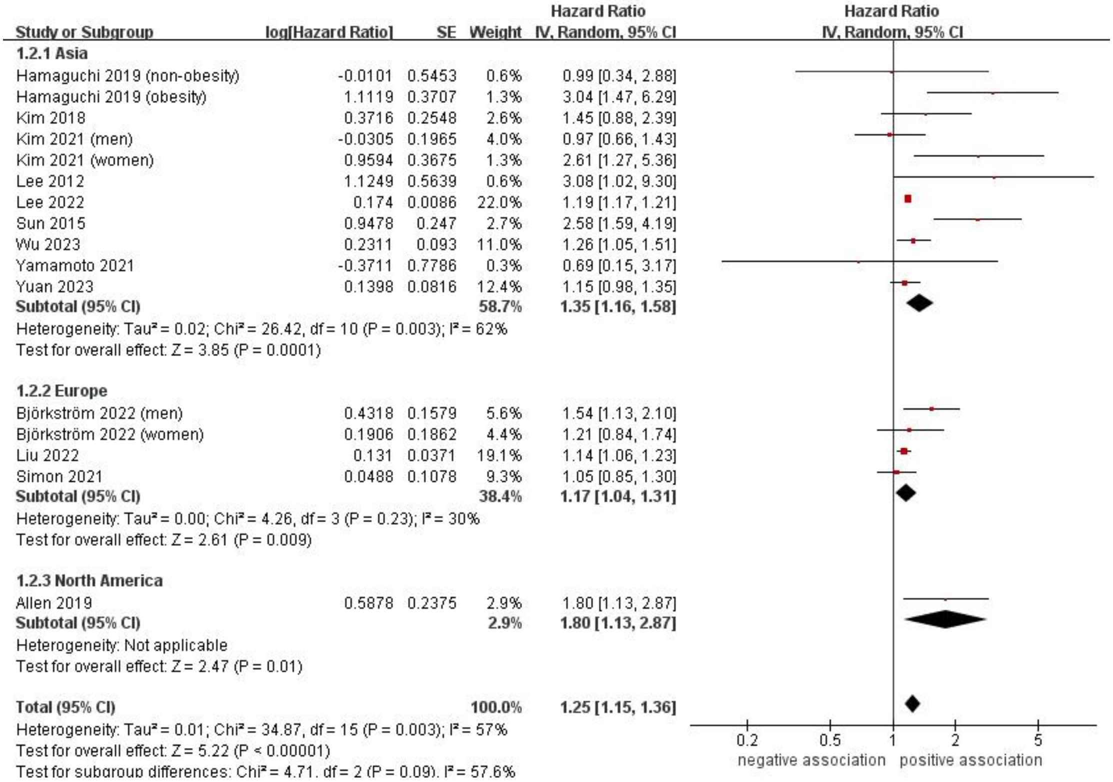

Figure S1. Forest plot of the association between MASLD and risk of CRC of subgroup analysis based on study location

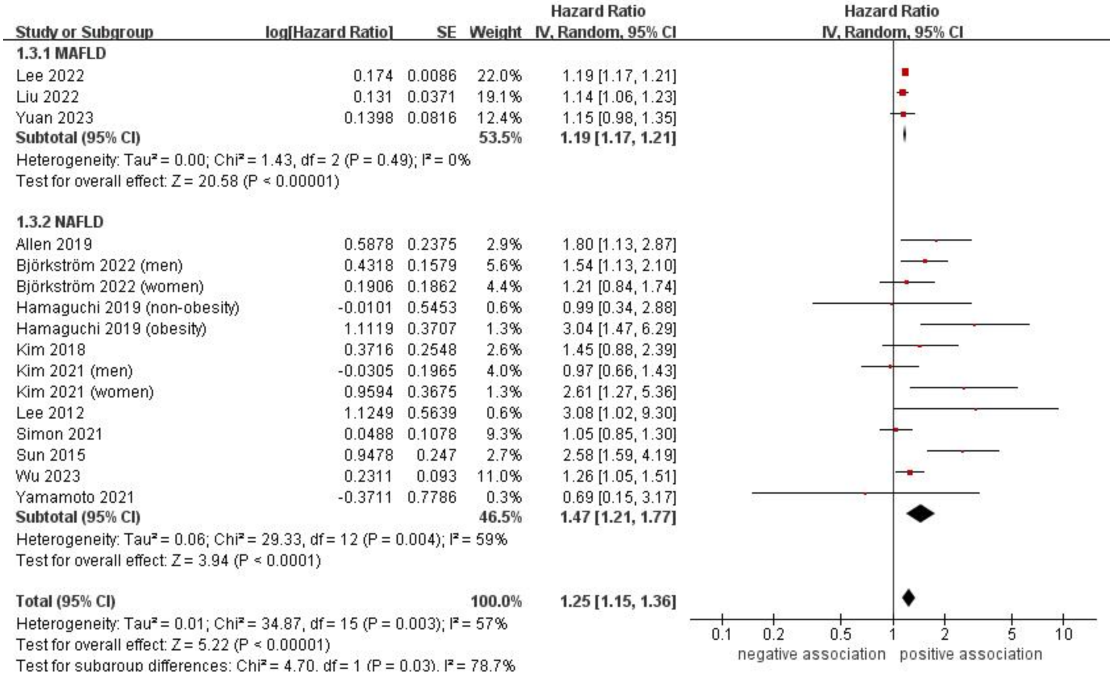

Figure S2. Forest plot of the association between MASLD and risk of CRC of subgroup analysis based on nomenclature of FLD

Study or Subgroup	log[Hazard Ratio]	SE	Weight	Hazard Ratio IV, Random, 95% CI
**1.4.1 USG**				
Hamaguchi 2019 (non-obesity)	-0.0101	0.5453	0.6%	0.99 [0.34, 2.88]
Hamaguchi 2019 (obesity)	1.1119	0.3707	1.3%	3.04 [1.47, 6.29]
Kim 2018	0.3716	0.2548	2.6%	1.45 [0.88, 2.39]
Kim 2021 (men)	-0.0305	0.1965	4.0%	0.97 [0.66, 1.43]
Kim 2021 (women)	0.9594	0.3675	1.3%	2.61 [1.27, 5.36]
Lee 2012	1.1249	0.5639	0.6%	3.08 [1.02, 9.30]
Yamamoto 2021	-0.3711	0.7786	0.3%	0.69 [0.15, 3.17]
Yuan 2023	0.1398	0.0816	12.4%	1.15 [0.98, 1.35]
**Subtotal (95% CI)**			**23.0%**	**1.45 [1.07, 1.97]**
Heterogeneity: Tau² = 0.09; Chi² = 16.07, df = 7 (P = 0.02); I² = 56%				
Test for overall effect: Z = 2.38 (P = 0.02)				
**1.4.2 FLI**				
Lee 2022	0.174	0.0086	22.0%	1.19 [1.17, 1.21]
Liu 2022	0.131	0.0371	19.1%	1.14 [1.06, 1.23]
**Subtotal (95% CI)**			**41.1%**	**1.18 [1.15, 1.22]**
Heterogeneity: Tau² = 0.00; Chi² = 1.27, df = 1 (P = 0.26); I² = 22%				
Test for overall effect: Z = 10.99 (P < 0.00001)				
**1.4.3 ICD codes**				
Allen 2019	0.5878	0.2375	2.9%	1.80 [1.13, 2.87]
Björkström 2022 (men)	0.4318	0.1579	5.6%	1.54 [1.13, 2.10]
Björkström 2022 (women)	0.1906	0.1862	4.4%	1.21 [0.84, 1.74]
Sun 2015	0.9478	0.247	2.7%	2.58 [1.59, 4.19]
Wu 2023	0.2311	0.093	11.0%	1.26 [1.05, 1.51]
**Subtotal (95% CI)**			**26.5%**	**1.52 [1.21, 1.92]**
Heterogeneity: Tau² = 0.04; Chi² = 9.49, df = 4 (P = 0.05); I² = 58%				
Test for overall effect: Z = 3.56 (P = 0.0004)				
**1.4.4 Liver biopsy**				
Simon 2021	0.0488	0.1078	9.3%	1.05 [0.85, 1.30]
**Subtotal (95% CI)**			**9.3%**	**1.05 [0.85, 1.30]**
Heterogeneity: Not applicable				
Test for overall effect: Z = 0.45 (P = 0.65)				
**Total (95% CI)**			**100.0%**	**1.25 [1.15, 1.36]**
Heterogeneity: Tau² = 0.01; Chi² = 34.87, df = 15 (P = 0.003); I² = 57%				
Test for overall effect: Z = 5.22 (P < 0.00001)				
Test for subgroup differences: Chi² = 7.48, df = 3 (P = 0.06), I² = 59.9%				

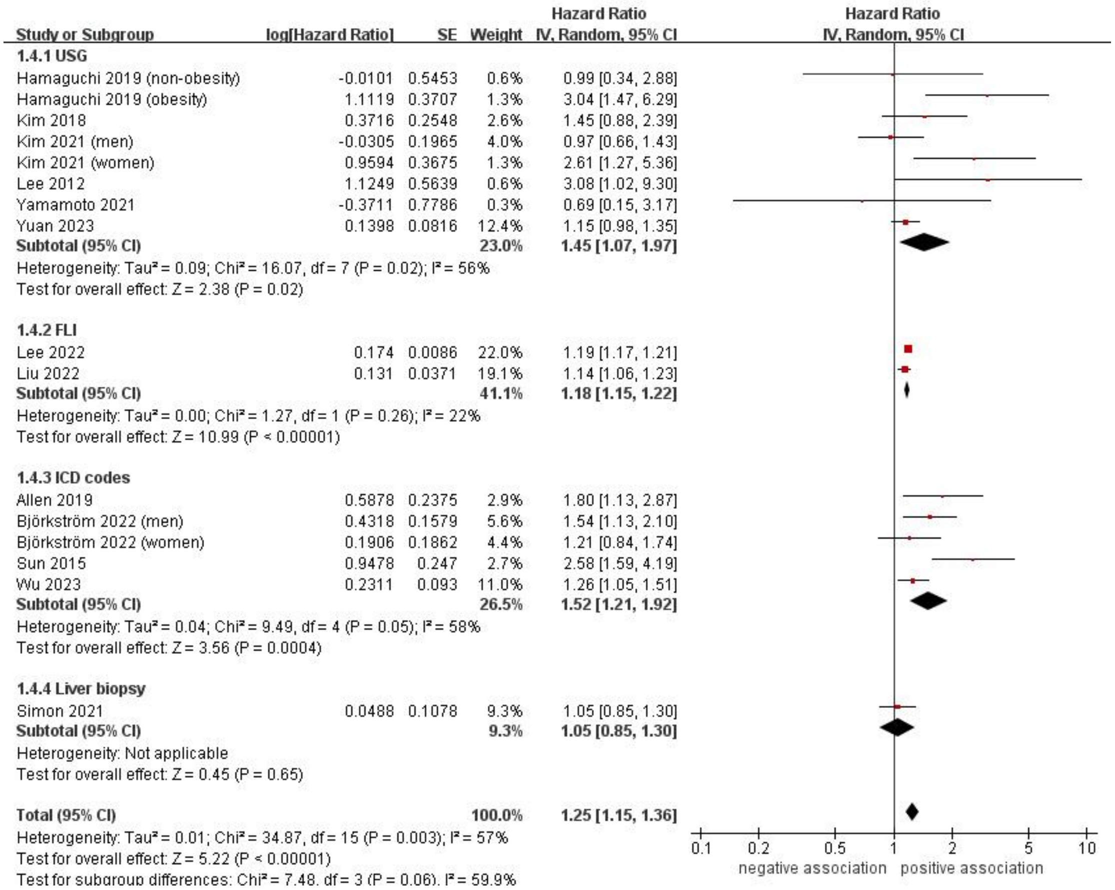

Figure S3. Forest plot of the association between MASLD and risk of CRC of subgroup analysis based on confirmation of FLD

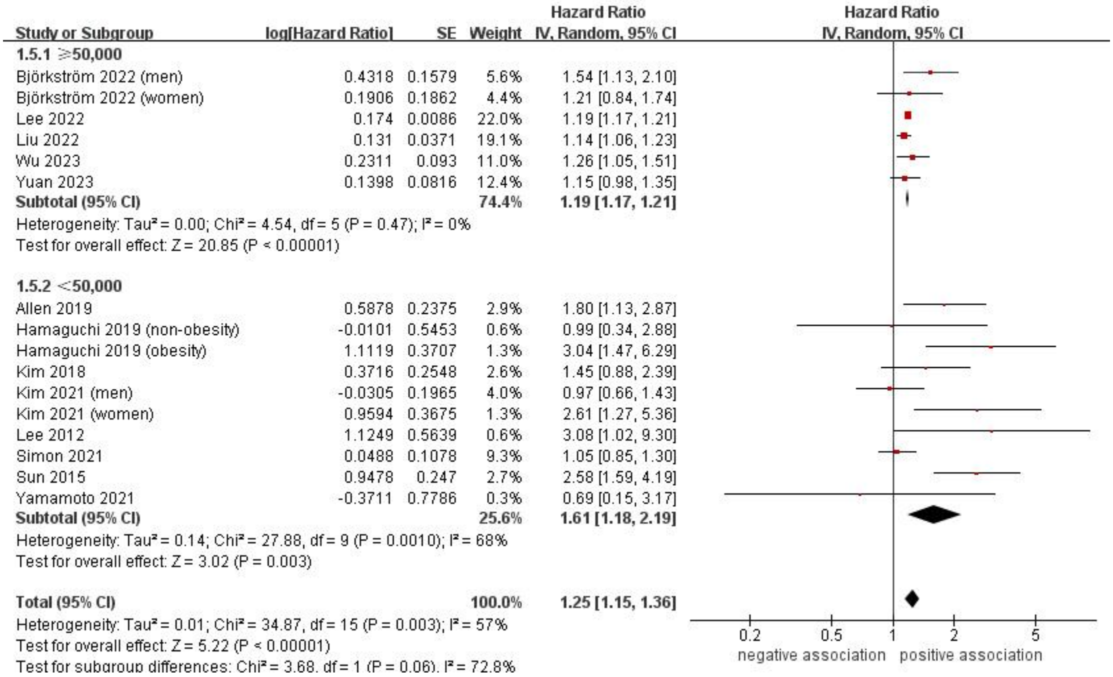

Figure S4. Forest plot of the association between MASLD and risk of CRC of subgroup analysis based on sample size

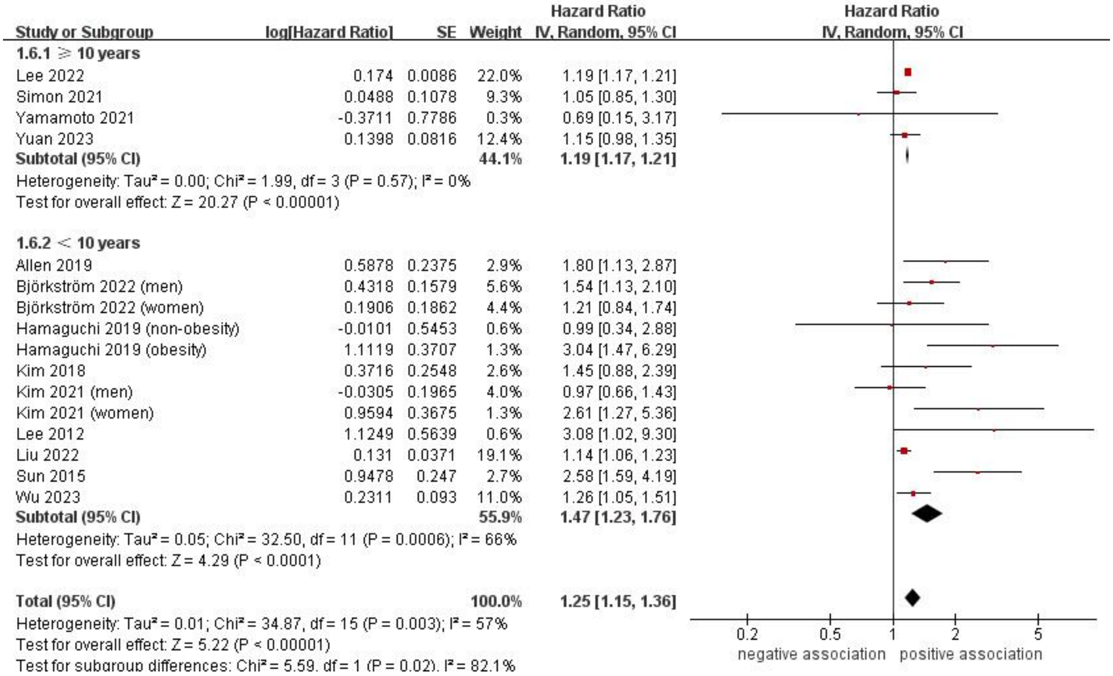

Figure S5. Forest plot of the association between MASLD and risk of CRC of subgroup analysis based on follow-up time

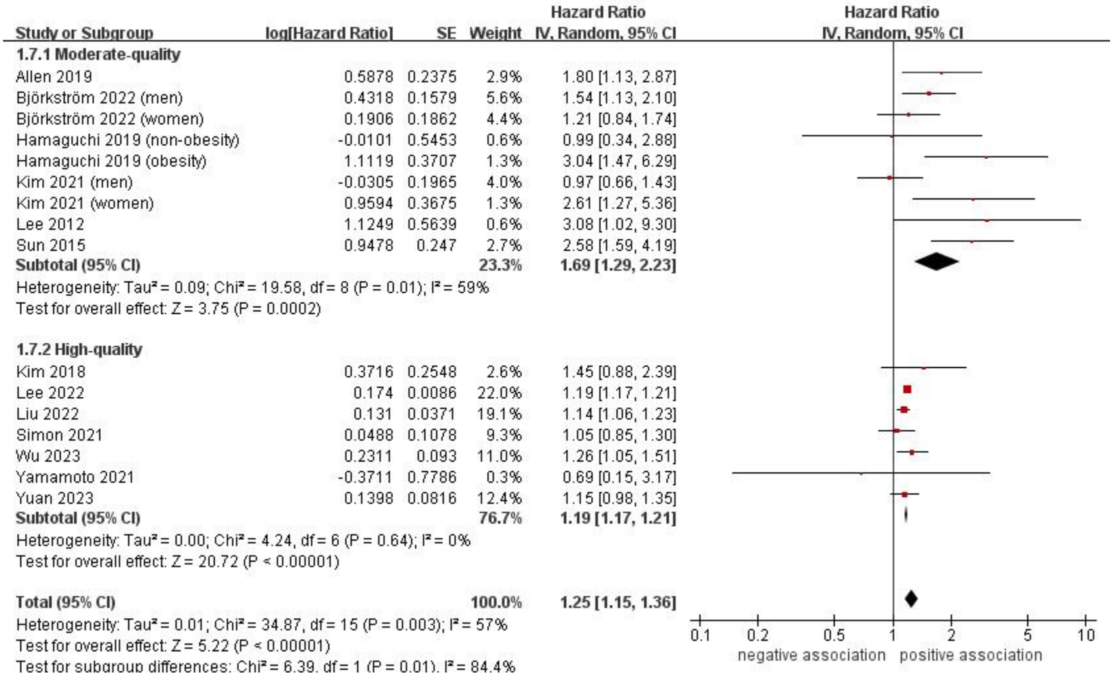

Study or Subgroup	log[Hazard Ratio]	SE	Weight	Hazard Ratio IV, Random, 95% CI
**1.7.1 Moderate-quality**				
Allen 2019	0.5878	0.2375	2.9%	1.80 [1.13, 2.87]
Björkström 2022 (men)	0.4318	0.1579	5.6%	1.54 [1.13, 2.10]
Björkström 2022 (women)	0.1906	0.1862	4.4%	1.21 [0.84, 1.74]
Hamaguchi 2019 (non-obesity)	-0.0101	0.5453	0.6%	0.99 [0.34, 2.88]
Hamaguchi 2019 (obesity)	1.1119	0.3707	1.3%	3.04 [1.47, 6.29]
Kim 2021 (men)	-0.0305	0.1965	4.0%	0.97 [0.66, 1.43]
Kim 2021 (women)	0.9594	0.3675	1.3%	2.61 [1.27, 5.36]
Lee 2012	1.1249	0.5639	0.6%	3.08 [1.02, 9.30]
Sun 2015	0.9478	0.247	2.7%	2.58 [1.59, 4.19]
**Subtotal (95% CI)**			**23.3%**	**1.69 [1.29, 2.23]**
Heterogeneity: Tau² = 0.09; Chi² = 19.58, df = 8 (P = 0.01); I² = 59%				
Test for overall effect: Z = 3.75 (P = 0.0002)				
**1.7.2 High-quality**				
Kim 2018	0.3716	0.2548	2.6%	1.45 [0.88, 2.39]
Lee 2022	0.174	0.0086	22.0%	1.19 [1.17, 1.21]
Liu 2022	0.131	0.0371	19.1%	1.14 [1.06, 1.23]
Simon 2021	0.0488	0.1078	9.3%	1.05 [0.85, 1.30]
Wu 2023	0.2311	0.093	11.0%	1.26 [1.05, 1.51]
Yamamoto 2021	-0.3711	0.7786	0.3%	0.69 [0.15, 3.17]
Yuan 2023	0.1398	0.0816	12.4%	1.15 [0.98, 1.35]
**Subtotal (95% CI)**			**76.7%**	**1.19 [1.17, 1.21]**
Heterogeneity: Tau² = 0.00; Chi² = 4.24, df = 6 (P = 0.64); I² = 0%				
Test for overall effect: Z = 20.72 (P < 0.00001)				
**Total (95% CI)**			**100.0%**	**1.25 [1.15, 1.36]**
Heterogeneity: Tau² = 0.01; Chi² = 34.87, df = 15 (P = 0.003); I² = 57%				
Test for overall effect: Z = 5.22 (P < 0.00001)				
Test for subgroup differences: Chi² = 6.39, df = 1 (P = 0.01), I² = 84.4%				

Figure S6. Forest plot of the association between MASLD and risk of CRC of subgroup analysis based on study quality

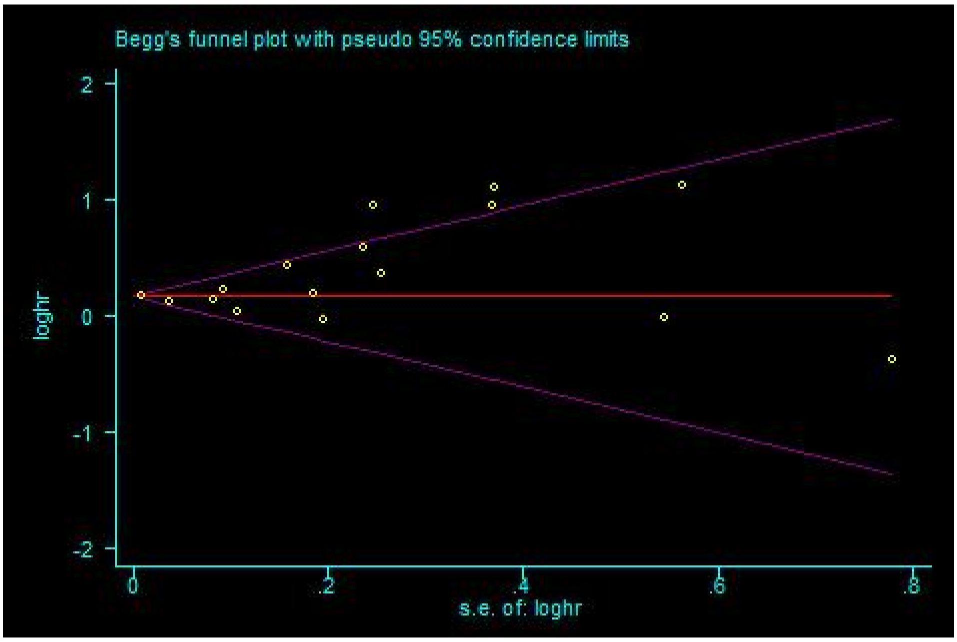

Figure S7. The Begg's funnel plot for the association between MASLD and risk of CRC
